# PSPC1 is a potential prognostic marker for hormone-dependent breast cancer patients and modulates RNA processing of *ESR1* and *SCFD2*

**DOI:** 10.1038/s41598-022-13601-7

**Published:** 2022-06-09

**Authors:** Toshihiko Takeiwa, Kazuhiro Ikeda, Takashi Suzuki, Wataru Sato, Kaori Iino, Yuichi Mitobe, Hidetaka Kawabata, Kuniko Horie, Satoshi Inoue

**Affiliations:** 1 Division of Systems Medicine and Gene Therapy, Saitama Medical University, 1397-1 Yamane, Hidaka, Saitama 350-1241 Japan; 2 Department of Systems Aging Science and Medicine, Tokyo Metropolitan Institute of Gerontology, 35-2 Sakae-cho, Itabashi-ku, Tokyo, 173-0015 Japan; 3 Department of Anatomic Pathology, Tohoku University Graduate School of Medicine, Sendai, Miyagi Japan; 4 Department of Breast and Endocrine Surgery, Toranomon Hospital, Tokyo, Japan

**Keywords:** Cancer, Oncology

## Abstract

Breast cancer is the most common cancer type among women worldwide. The majority of breast cancer expresses estrogen receptor (ER) and endocrine therapy is a standard treatment of ER-positive breast cancer. However, development of the therapy resistance is still a major challenge and thus new therapeutic approaches are needed. Here we show that an RNA-binding protein, PSPC1, play a crucial role in ER-positive breast cancer growth through post-transcriptional gene regulation. We showed that siRNA-mediated PSPC1 silencing suppressed the proliferation of ER-positive breast cancer cells. Strong immunoreactivity (IR) of PSPC1 was correlated with poor prognosis for ER-positive breast cancer patients. Using immunoprecipitation, RNA-immunoprecipitation (RIP) and quantitative PCR (qPCR) experiments, we showed that PSPC1 interacted with PSF and was involved in post-transcriptional regulation of PSF target genes, *ESR1* and *SCFD2*. Strong SCFD2 IR was correlated with poor prognosis for ER-positive breast cancer patients and combinations of PSPC1, PSF, and SCFD2 IRs were potent prognostic factors. Moreover, we identified *DDIAS* and *MYBL1* as SCFD2 downstream target genes using microarray analysis, and finally showed that *SCFD2* silencing suppressed tamoxifen-resistant breast tumor growth in vivo. These results indicated that PSPC1 and SCFD2 axis could be a promising target in the clinical management of the disease.

Breast cancer is the most common cancer type with the highest incidence and mortality rates among women worldwide^1,2^. The majority (70–80%) of breast cancers are hormone-dependent with expression of estrogen receptor (ER) and its target progesterone receptor (PR)^3^. Although they initially respond to endocrine therapy such as ER antagonist tamoxifen^3^, acquired endocrine therapy resistance is often occurred in hormone-dependent tumors as a risk of distant recurrence of ~ 10% during years 5–20 even among patients with small, node-negative, low-grade tumor^4^. Novel therapeutic targets are therefore required to overcome the endocrine therapy resistance.

RNA-binding proteins (RBPs) are key players in gene expression, particularly at post-transcriptional levels^5^. RBPs regulate the cellular dynamics of RNAs, such as RNA processing, localization, and decay. Recent studies demonstrated that RBPs play an important role in the development and progression of various cancers, thus suggesting that RBPs may serve as new therapeutic targets for different cancers^6–9^. We previously demonstrated that PSF, an RBP belonging to *Drosophila* behavior human splicing (DBHS) family^10^, exerts oncogenic roles in breast and prostate cancers through post-transcriptional gene regulation^11,12^. PSF contributes to RNA processing of *ESR1* and *SCFD2* in ER-positive breast cancer, which promotes the cancer progression^12^. We also showed that PSF upregulates spliceosome gene expression, which plays a role in the splicing of androgen receptor (AR) in hormone-refractory prostate cancer^11^. Recent studies reported that another DBHS family RBP PSPC1 acts as a transcriptional regulator in cancers, facilitating cancer stemness, epithelial-to-mesenchymal transition (EMT), and metastasis in breast and lung cancers and hepatocellular carcinoma (HCC)^13,14^. PSPC1-mediated post-transcriptional gene regulation in cancer, however, remains to be elucidated.

Here, we demonstrate that PSPC1 is associated with poor prognosis in ER-positive breast cancer patients and involved in the post-transcriptional regulation of *ESR1* and *SCFD2*, both were previously defined as PSF target genes by our previous study^12^. We here identified anti-apoptotic genes *DDIAS* and *MYBL1* as downstream target genes of SCFD2 in ER-positive breast cancer cells. We further defined that combinations of strong intensities for PSPC1, SCFD2, and PSF immunostaining could be potent prognostic factors for ER-positive breast cancer patients.

## Results

### PSPC1 knockdown represses ER-positive breast cancer cell proliferation

**Figure 1:**
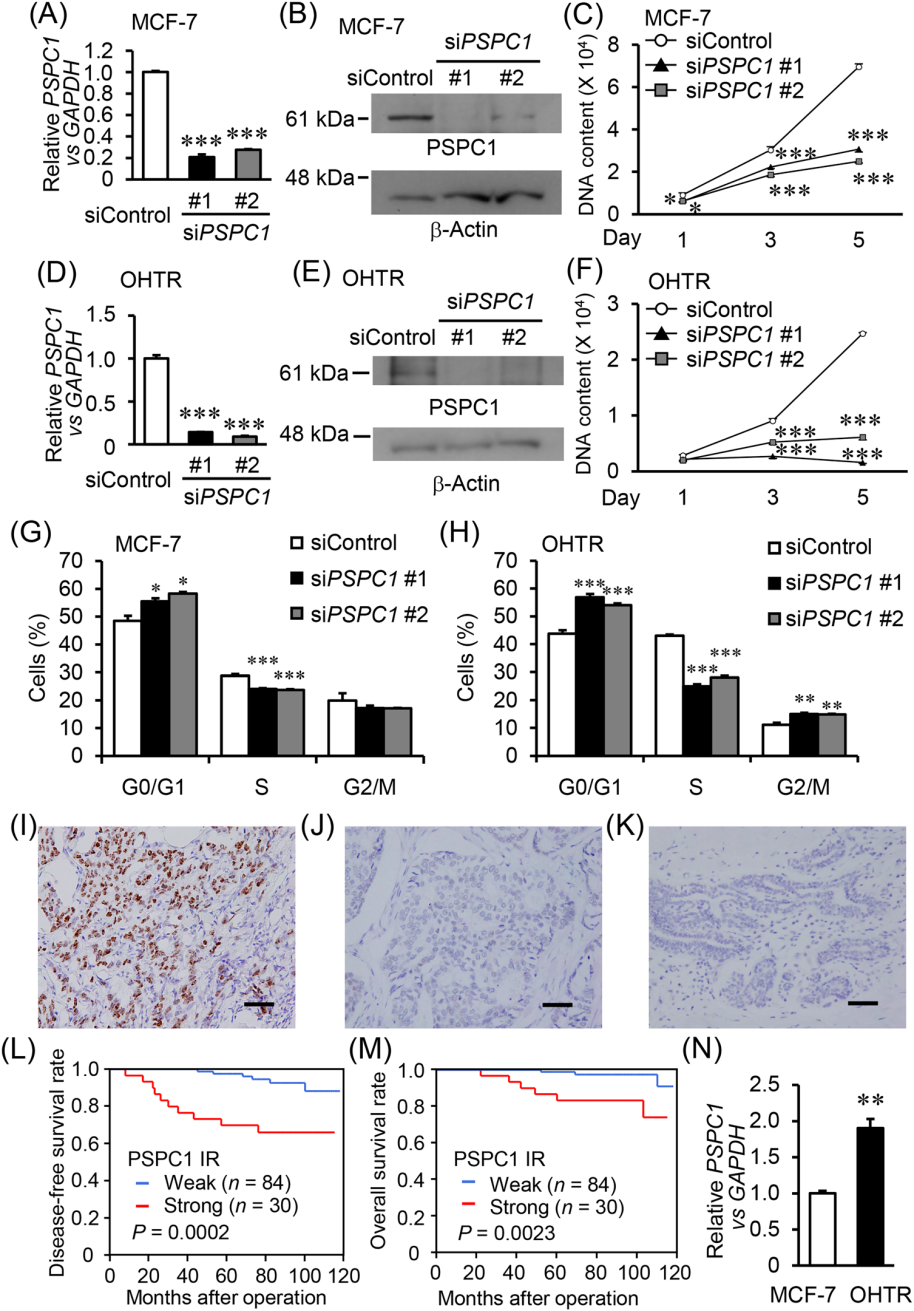
PSPC1 is associated with ER-positive breast cancer growth and poor prognosis for ER-positive breast cancer patients. (**A**,**D**) *PSPC1* mRNA levels in MCF-7 (**A**) and its 4-hydroxytamoxifen (OHT)-resistant derivative OHTR (**D**) cells transfected with control (siControl) or PSPC1-specific (si*PSPC1* #1 and #2) siRNAs (10 nM) analyzed by qRT-PCR. Relative *PSPC1* mRNA level was normalized to *GAPDH* mRNA level in each sample and presented as mean fold change ± SEM compared with siControl (*n* = 3). (**B**,**E**) PSPC1 protein levels in MCF-7 (**B**) and OHTR (**E**) cells transfected with indicated siRNAs (10 nM) analyzed by Western blotting. β-Actin was used as a loading control. Unprocessed original scans of the blots are shown in Supplementary Fig. S3. (**C**,**F**) DNA assay for MCF-7 (**C**) and OHTR (**F**) cells treated with the indicated siRNAs (10 nM). Data are presented as mean value ± SEM (*n* = 5). (**G**,**H**) Cell-cycle analysis in MCF-7 (**G**) and OHTR (**H**) cells after treatment with indicated siRNAs using flow cytometry. Percentages of cell populations in G0/G1-, S-, and G2/M-phases are shown. Data are presented as mean value ± SEM (*n* = 3). Original flow cytometry data are shown in Supplementary Fig. S4. (**I**–**K**) Representative images of ER-positive breast cancer tissue sections with strong (**I**) and weak (**J**) PSPC1 immunoreactivity (IR), and benign breast ducts (**K**). Scale bar, 50 μm. (**L**,**M**) Kaplan–Meier plots for disease-free (**L**) and overall (**M**) survivals of ER-positive breast cancer patients with weak and strong PSPC1 IR (*n* = 84 and 30, respectively). (**N**) *PSPC1* mRNA levels in MCF-7 and OHTR cells analyzed by qRT-PCR. Relative *PSPC1* mRNA level was normalized to *GAPDH* mRNA level in each sample and presented as mean fold change ± SEM (*n* = 3). *P < 0.05; **P < 0.01; ***P < 0.001, two-way ANOVA performed in (**A**,**C**,**D**,**F**,**G**,**H**). **P < 0.01, Student’s *t*-test performed in (**N**).

To explore the role of PSPC1 in ER-positive breast cancer cells, we examined whether PSPC1 contributes to ER-positive breast cancer cell growth using two distinct siRNAs targeting PSPC1. The siRNAs efficiently downregulated PSPC1 expression in human ER-positive breast cancer MCF-7 cells and its 4-hydroxytamoxifen (OHT)-resistant OHTR cells^15^ at the mRNA and protein levels (Fig. 1A,B,D,E), and significantly suppressed cell proliferation (Fig. 1C,F). We also demonstrated that PSPC1 silencing in MCF-7 and OHTR cells increased the percentage of cells in the G0/G1-phase and decreased the percentage of those in the S-phase (Fig. 1G,H).

### PSPC1 is associated with poor prognosis for ER-positive breast cancer patients

Next, to examine the clinical significance of PSPC1 in breast cancer, we performed immunohistochemical analysis of PSPC1 in 114 ER-positive breast cancer samples. PSPC1 signals were strong in 30 tumor samples, whereas weak in 84 tumor samples and normal mammary tissues (Fig. 1I–K). In correlation analysis between PSPC1 status and clinicopathological parameters, *P* values in correlation were 0.080 and 0.089 for stage and PSF IR status that we previously determined^12^, respectively (Table 1). In Kaplan–Meier survival analysis, PSPC1 strong IR was significantly correlated with shorter disease-free survival (Fig. 1L, *P* = 0.0002) and overall survival (Fig. 1M, *P* = 0.0023) of the patients. Intriguingly, univariate and multivariate analyses indicated that PSPC1 IR status is an independent prognostic factor of ER-positive breast cancer (Table 2). Notably, we observed that *PSPC1* expression was elevated in tamoxifen-resistant OHTR cells compared with parental MCF-7 cells (Fig. 1N).

**Table 1:** Association between PSPC1 status and clinicopathological parameters in 114 breast cancer.

Status	PSPC1 status		*P *value
	Weak (*n* = 84)	Strong (*n* = 30)	
Age (year)^a^	53.4 ± 10.0	51.4 ± 13.6	0.39
Body weight (kg)^a^	55.0 ± 8.9	52.3 ± 5.7	0.12
BMI^a^	21.9 ± 3.2	21.8 ± 3.7	0.85
**Stage**			
I	48	10	
II	32	18	
III	4	2	*0.080*
**Pathological T factor (pT)**			
pT1	56	16	
pT2–4	28	14	0.19
**Pathological N factor (pN)**			
pN0–1	67	20	
pN2–3	17	10	0.15
**Histological grade**			
1–2	75	24	
3	9	6	0.20
**Lymphovascular infiltration (LVI)**			
Negative	50	13	
Positive	34	17	0.34
ER labeling index (%)^a^	68.6 ± 26.1	72.9 ± 24.2	0.43
**PR status**			
Negative	12	6	
Positive	72	24	0.46
**HER2 status**			
Negative	76	25	
Positive	8	5	0.29
**PSF status**			
Weak	46	11	
Strong	38	19	*0.089*

0.05 ≤ *P* value < 0.10 was considered borderline significant, and is listed in italics.

^a^Data are presented as mean ± SD. All other values represent the number of cases.

**Table 2:** Univariate and multivariate analyses of disease-free and overall survivals in 114 ER-positive breast cancer patients.

Variable	Univariate	Multivariate	
	*P* value	*P* value	Relative risk (95% CI)
**Disease-free survival**			
PSPC1 (weak/strong)	**0.0010**^†^	**0.011**	3.9 (1.4–11.3)
pN (N0,1/N2)	**0.0021**^†^	**0.049**	2.9 (1.0–8.5)
pT (pT1/pT2–4)	**0.0077**^†^	*0.053*	3.2 (1.0–10.1)
SCFD2 (weak/strong)	**0.033**^†^	*0.083*	2.6 (0.9–7.4)
PR (negative/positive)	0.24		
HER2 status (negative/positive)	0.44		
PSF (weak/strong)	0.49		
Histological grade	0.51		
**Overall survival**			
PSPC1 (weak/strong)	**0.0079**^†^	**0.0053**	10.5 (2.0–54.5)
pN (N0,1 /N2)	**0.0093**^†^	**0.0053**	8.3 (1.9–37.1)
SCFD2 (weak/strong)	**0.021**^†^	*0.075*	4.4 (0.9–22.2)
PSF (weak/strong)	**0.035**^†^	**0.031**	10.8 (1.3–93.5)
PR (negative/positive)	0.56		
pT (pT1/pT2–4)	0.17		
HER2 status (negative/positive)	0.20		
Histological grade	0.79		

Statistical analysis was evaluated by a proportional hazard model (Cox).

*P* value < 0.05 and 0.05 ≤ *P* value < 0.10 were considered significant and borderline significant, and were listed in bold and italic, respectively.

*95% CI* 95% confidence interval.

^†^Significant (*P* < 0.05) values were examined in the multivariate analyses in this study.

### PSPC1 modulates post-transcriptional regulation of PSF target genes *ESR1* and *SCFD2*

**Figure 2:**
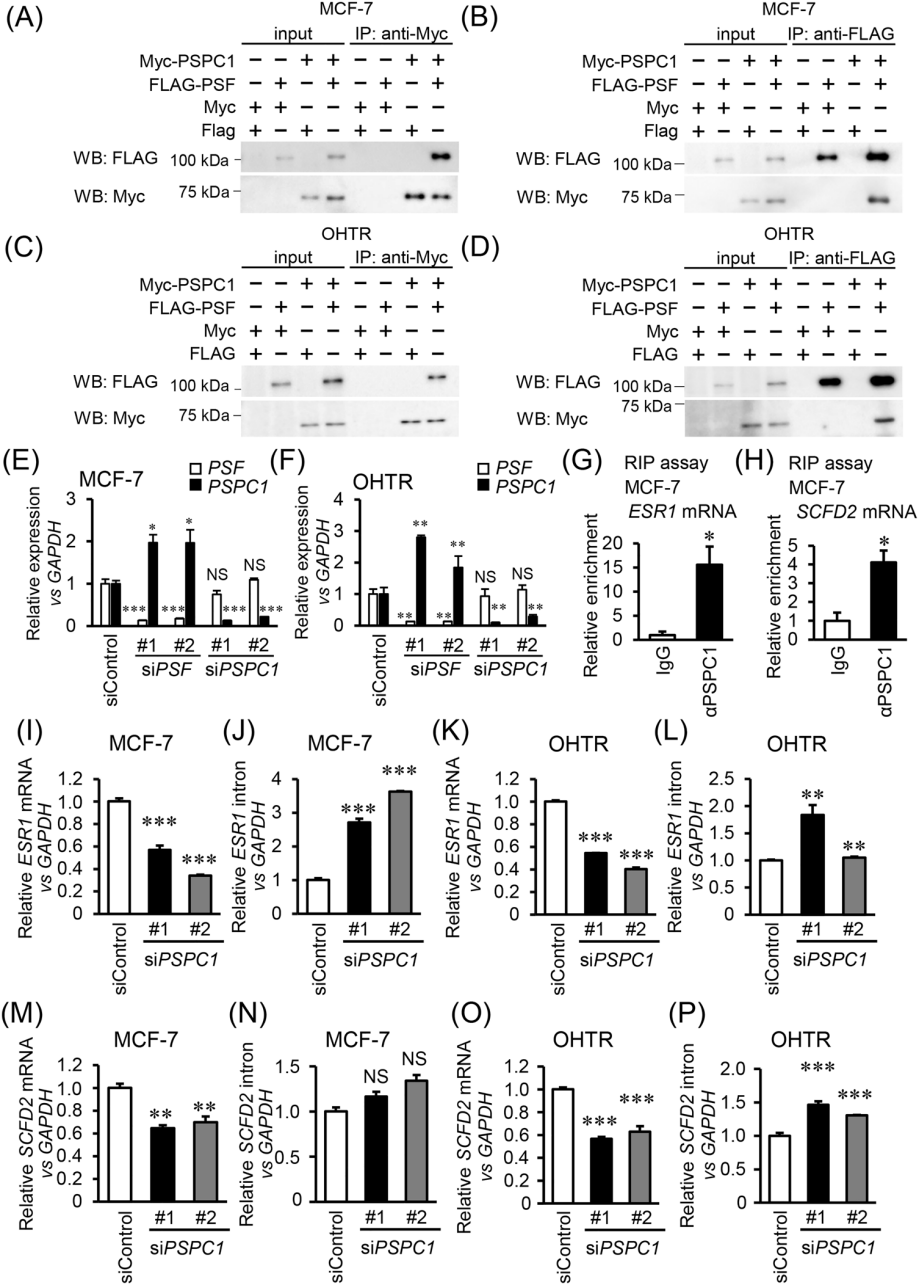
PSPC1 post-transcriptionally modulates *ESR1* and *SCFD2* expression. (**A**,**B**) MCF-7 cells were transfected with plasmids encoding indicated proteins and cell lysates were prepared 24 h after transfection. Myc-tagged PSPC1 (Myc-PSPC1) and FLAG-tagged PSF (FLAG-PSF) were immunoprecipitated with Anti-c-Myc Agarose (**A**) or ANTI-FLAG M2 Affinity Gel (**B**), respectively. Precipitated proteins were eluted with 2 × SDS sample buffer and subjected to Western blot analysis with anti-Myc and FLAG antibodies. Unprocessed original scans of the blots are shown in Supplementary Fig. S3. (**C**,**D**) The same experiments as (**A**) and (**B**) were performed using OHTR cells. Unprocessed original scans of the blots are shown in Supplementary Fig. S3. (**E**,**F**) *PSF* and *PSPC1* mRNA levels in MCF-7 (**E**) and OHTR (**F**) cells transfected with siRNAs for *PSF* and *PSPC1* for 48 h were analyzed by qRT-PCR. Relative *PSF* and *PSPC1* mRNA levels were normalized to *GAPDH* mRNA level in each sample and presented as mean fold change ± SEM (*n* = 3). *P < 0.05; **P < 0.01; ***P < 0.001; *NS* not significant, two-way ANOVA. (**G**,**H**) Interaction of PSPC1 with *ESR1* (**G**) and *SCFD2* (**H**) mRNAs in MCF-7 cells. RNA-immunoprecipitation (RIP) assay was performed using anti-PSPC1 antibody or normal mouse IgG. *ESR1* and *SCFD2* mRNA levels were analyzed by qRT-PCR. Each mRNA level was normalized to *GAPDH* mRNA level in each sample. The fold enrichment relative to IgG was shown as mean and SEM (*n* = 3). *P < 0.05, Student’s *t*-test. (**I**–**P**), Effects of PSPC1 knockdown on *ESR1* mRNA (**I**,**K**) and intron (**J**,**L**) or *SCFD2* mRNA (**M**,**O**) and intron (**N**,**P**) levels in MCF-7 and OHTR cells analyzed by qRT-PCR. Relative expression levels of these RNAs were normalized to *GAPDH* mRNA level in each sample and presented as mean fold change ± SEM compared with siControl (*n* = 3). **P < 0.01; ***P < 0.001; *NS* not significant, two-way ANOVA.

Previous studies have shown that PSPC1 structurally interacts with PSF^16^. Consistent to these findings, we observed the interaction of these proteins in MCF-7 and OHTR breast cancer cells by immunoprecipitation assay (Fig. 2A–D). siRNA-mediated silencing experiments in these cells showed that *PSF* expression was not affected by *PSPC1* silencing, while *PSPC1* expression was elevated by *PSF* knockdown (Fig. 2E,F). To examine if PSPC1 cooperatively functions with PSF in ER-positive breast cancer cells, we next analyzed whether PSPC1 affects the post-transcriptional regulation of *ESR1* and *SCFD2,* which we previously defined as PSF downstream targets^12^. RNA-immunoprecipitation (RIP) assay indicated that *ESR1* and *SCFD2* mRNAs were specifically precipitated by immunoprecipitation of PSPC1, indicating that PSPC1 protein functionally associates with these mRNAs (Fig. 2G,H). The qRT-PCR analysis with primer sets for *ESR1* and *SCFD2* mRNAs demonstrated that PSPC1-specific siRNAs substantially repressed *ESR1* and *SCFD2* expression compared with control siRNA (Fig. 2I,K,M,O). However, qRT-PCR with another primer set for intron reveals that PSPC1-specific siRNAs increased or had no effect on intron-containing pre-mRNAs of *ESR1* and *SCFD2*, implicating a discordant effect on pre-mRNA maturation or splicing (Fig. 2J,L,N,P). We further performed siRNA-mediated double knockdown experiments for PSPC1/PSF. While PSPC1 single knockdown significantly impaired MCF-7 and OHTR cell proliferation, double knockdown of PSPC1/PSF markedly suppressed cell proliferation (Supplementary Fig. S1), suggesting possible cooperative functions of PSPC1 with PSF.

### SCFD2 is associated with poor prognosis for ER-positive breast cancer patients

**Figure 3:**
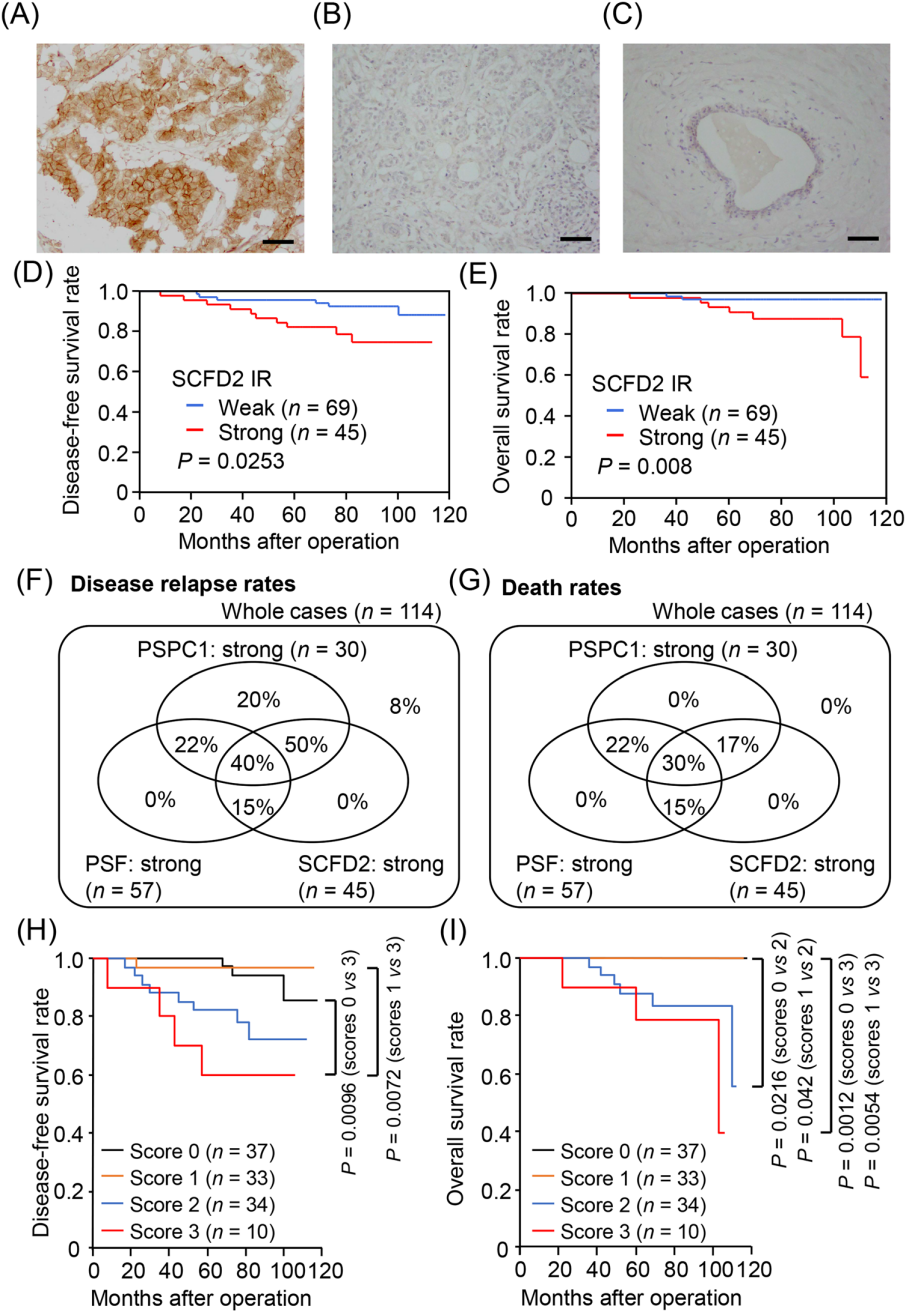
SCFD2 is a potential prognostic factor of ER-positive breast cancer patients and combinations of PSPC1/PSF/SCFD2 IRs are potently associated with poor prognosis in ER-positive breast cancer patients. (**A**–**C**) Representative images of ER-breast tumor sections with strong (**A**) and weak (**B**) SCFD2 IR and benign breast duct (**C**). Scale bar, 50 μm. (**D**,**E**) Kaplan–Meier plots for disease-free (**D**) and overall (**E**) survivals of ER-positive breast cancer patients with weak and strong SCFD2 IR (*n* = 69 and 45, respectively). (**F**,**G**) Disease relapse rates (**F**) and death rates (G) among ER-positive breast cancer patients categorized by PSPC1/PSF/SCFD2 IR status. (**H**,**I**) Kaplan–Meier plots for disease-free (**H**) and overall (**I**) survivals of ER-positive breast cancer patients categorized by PSPC1/PSF/SCFD2 IR scores. Score 0: weak IR for all three proteins, score 1: strong IR for any single protein of PSPC1/PSF/SCFD2, score 2: strong IR for any two proteins of PSPC1/PSF/SCFD2, and score 3: strong IR for all three proteins. For multiple comparisons, significance is assessed at *P* < 0.05 following Bonferroni correction.

We demonstrated that *SCFD2* mRNA levels were associated with poor prognosis for ER-positive breast cancer patients using Kaplan–Meier plotter database in the previous study^12^. Immunohistochemical analysis was performed to reveal the role of SCFD2 protein in clinical samples, and SCFD2 IR was strong in 45 cases whereas weak in 69 cases among the present 114 cases (Fig. 3A,B). Normal mammary tissues showed weak or negligible SCFD2 IR (Fig. 3C). SCFD2 status was positively correlated with histological grade, PSF status, and HER2 status, and had a tendency of correlation (*P* < 0.1) with LVI and PSPC1 IR status (Table 3). In Kaplan–Meier survival analysis, strong SCFD2 IR was significantly associated with shorter disease-free survival (*P* = 0.0253) and overall survival (*P* = 0.008) of ER-positive breast cancer patients (Fig. 3D,E) Based on univariate and multivariate analyses, SCFD2 IR had a tendency to be an independent prognostic factor for disease-free and overall survivals in these patients (Table 2).

**Table 3:** Association between SCFD2 status and clinicopathological parameters in 114 breast cancer.

Status	SCFD2 status		*P *value
	Weak (*n* = 69)	Strong (*n* = 45)	
Age (year)^a^	51.8 ± 10.4	54.5 ± 11.9	0.20
Body weight (kg)^a^	55.0 ± 7.9	53.3 ± 8.8	0.28
BMI^a^	21.9 ± 3.6	21.9 ± 2.9	0.94
**Stage**			
I	37	21	
II	30	20	
III	2	4	0.35
**Pathological T factor (pT)**			
pT1	42	30	
pT2–4	27	15	0.53
**Pathological N factor (pN)**			
pN0–1	55	32	
pN2–3	14	13	0.29
**Histological grade**			
1–2	64	35	
3	5	10	**0.021**
**Lymphovascular infiltration (LVI)**			
Negative	43	20	
Positive	26	25	*0.061*
ER labeling index (%)^a^	68.7 ± 25.4	71.3 ± 26.1	0.59
**PR status**			
Negative	10	8	
Positive	59	37	0.64
**HER2 status**			
Negative	65	36	
Positive	4	9	**0.021**
**PSF status**			
Weak	42	15	
Strong	27	30	**0.0041**
**PSPC1 status**			
Weak	55	29	
Strong	14	16	*0.070*

*P* value < 0.05 and 0.05 ≤ *P* value < 0.10 were considered significant and borderline significant, and were listed in bold and italic, respectively.

^a^Data are presented as mean ± SD. All other values represent the number of cases.

### Combinations of PSPC1, PSF, and SCFD2 IRs are potent prognostic factors for ER-positive breast cancer

As PSPC1, PSF and SCFD2 IRs were shown to be potential prognostic factors for ER-positive breast cancer patients^12^, we further examined whether combinations of IRs of these proteins could efficiently predict the prognosis of ER-positive breast cancer patients. Among the 114 patients, those with strong IR of any two proteins tended to have higher rates of disease relapse and death compared with those with strong IR of any single protein alone (Fig. 3F,G). In particular, the disease relapse event was not observed among patients with strong PSF or SCFD2 IR alone, while it occurred in 15% of those with strong IR of PSF/SCFD2 (Fig. 3F). While the rate of disease relapse was 20% in patients with strong PSPC1 IR alone, it was much higher as 50% in those with strong IR of PSPC1/SCFD2 (Fig. 3F). It is notable that no death was observed in patients with strong IR of any single protein alone (Fig. 3G). The death rate was increased in patients with strong IR of PSPC1/PSF, PSPC1/SCFD2, and PSF/SCFD2 (22%, 17%, and 15%, respectively), and was 30% in those with strong IR of all three proteins (Fig. 3G). In Kaplan–Meier survival analysis, patients with tumors exhibiting score 2 IR of any two proteins were associated with shorter overall survival compared to those with tumors exhibiting score 0 IR of all proteins or score 1 IR of any single protein (*P* = 0.0216 or 0.042, respectively, corrected by Bonferroni method). Moreover, combinations of score 3 IR of all three proteins were significantly correlated with shorter disease-free survival compared to the score 0 or 1 IR (*P* = 0.0096 or 0.0072, respectively, corrected by Bonferroni method), and with overall survival compared to the score 0 or 1 IR (*P* = 0.0012 or 0.0054, respectively, corrected by Bonferroni method) (Fig. 3H,I). These results indicate that double or triple strong IRs of PSPC1/PSF/SCFD2 could be potent prognostic factors for ER-positive breast cancer patients.

### High expression of SCFD2 downstream target genes *DDIAS *and *MYBL1* is associated with poor prognosis in ER-positive breast cancer patients

**Figure 4:**
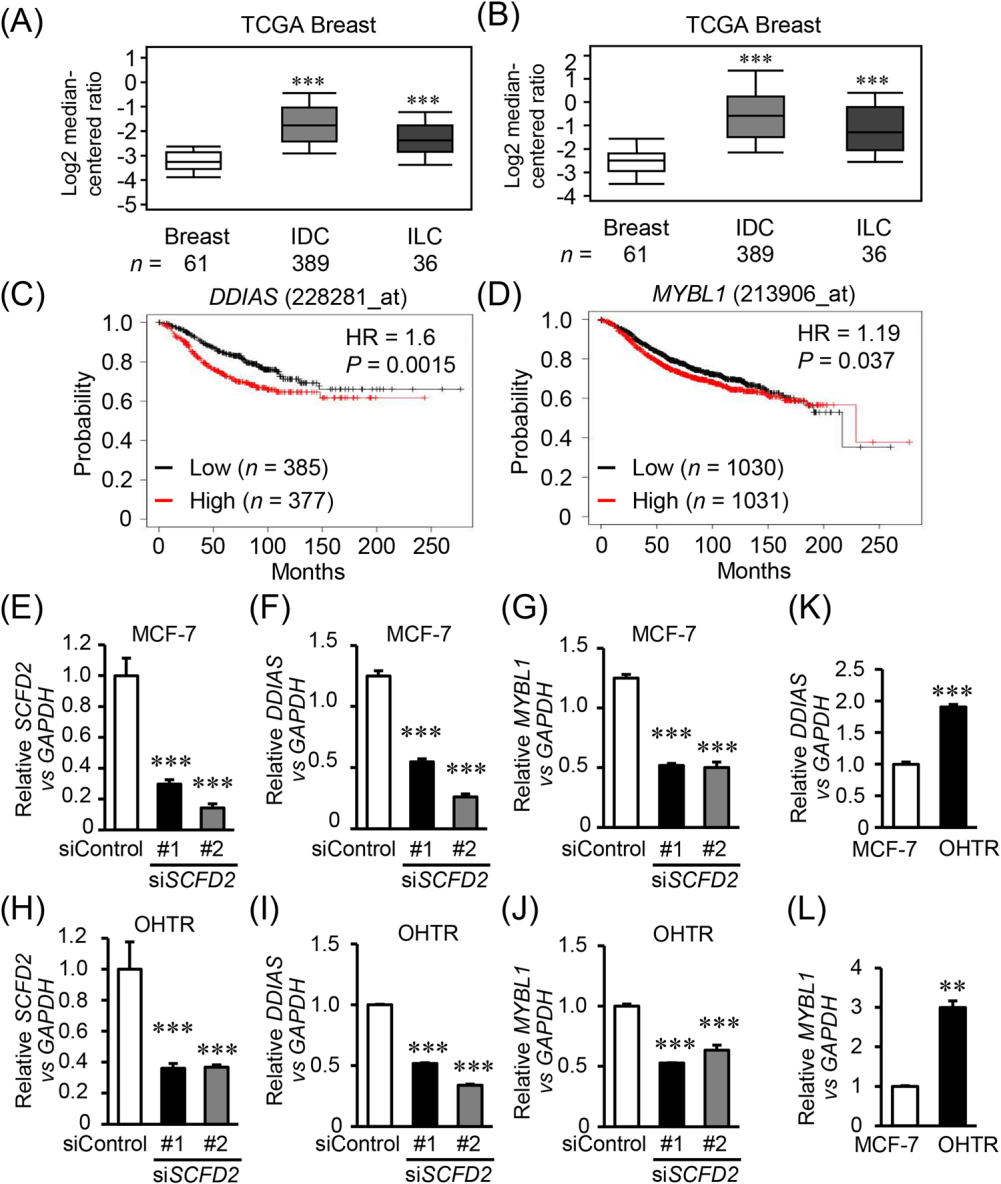
*DDIAS* and *MYBL1* are *SCFD2* downstream target genes and associated with worse prognosis in ER-positive breast cancer. (**A**,**B**) *DDIAS* (**A**) and *MYBL1* (**B**) mRNA expression in normal breast tissues (Breast), invasive ductal breast carcinoma (IDC), and invasive lobular breast carcinoma (ILC) based on TCGA breast cancer dataset retrieved from the Oncomine™ Platform (www.oncomine.org). (**C**,**D**) Relapse-free survival of ER-positive breast cancer patients with high or low mRNA expression of *DDIAS* (**C**) or *MYBL1* (**D**) analyzed by online Kaplan–Meier plotter (http://kmplot.com/). (**E**–**J**) *SCFD2* (**E**,**H**), *DDIAS* (**F**,**I**), and *MYBL1* (**G**,**J**) mRNA levels in MCF-7 and OHTR cells transfected with control (siControl) or SCFD2-specific (si*SCFD2* #1 and #2) siRNAs (10 nM) were analyzed by qRT-PCR. Relative expression levels of these mRNAs were normalized to *GAPDH* mRNA level in each sample and presented as mean fold ± SEM compared with siControl (*n* = 3). (**K**,**L**) Relative expression levels of *DDIAS* (**K**) and *MYBL1* (**L**) mRNAs in MCF-7 and OHTR cells were normalized to *GAPDH* mRNA level in each sample and presented as mean fold ± SEM compared with siControl (*n* = 3). **P < 0.01; ***P < 0.001, two-way ANOVA performed in Fig. 5E–J. **P < 0.01; ***P < 0.001, Student’s *t*-test performed in Fig. 5K,L .

To elucidate SCFD2-dependent signaling in ER-positive breast cancer, we further performed transcriptomic analysis in MCF-7 cells with or without SCFD2 knockdown. We picked up 150 genes most significantly repressed by SCFD2 siRNA versus control siRNA and further analyzed enriched pathways among the genes based on gene ontology (GO) terms using AmiGO2 database. “Cell cycle process” was most significantly associated with the genes (Supplementary Table S1 and Supplementary Fig. S2). Among cell cycle process-related genes, we found that *DDIAS* and *MYBL1* were particularly overexpressed in invasive ductal breast carcinoma (IDC) or invasive lobular breast carcinoma (ILC) samples than normal breast tissues in TCGA breast cancer dataset retrieved from the Oncomine™ Platform (Fig. 4A,B). Online Kaplan–Meier plotter (http://kmplot.com/) showed that high expression of *DDIAS* and *MYBL1* mRNAs in tumors was significantly associated with shorter relapse-free survival in ER-positive breast cancer patients (Fig. 4C,D). We found that both *DDIAS* and *MYBL1* were substantially downregulated in MCF-7 and OHTR cells treated with SCFD2 siRNAs (Fig. 4E–J). *DDIAS* and *MYBL1* expression levels were higher in OHTR cells compared to MCF-7 cells (Fig. 4K,L).

### SCFD2 silencing suppresses in vivo tumor growth of tamoxifen-resistant breast cancer cells

**Figure 5:**
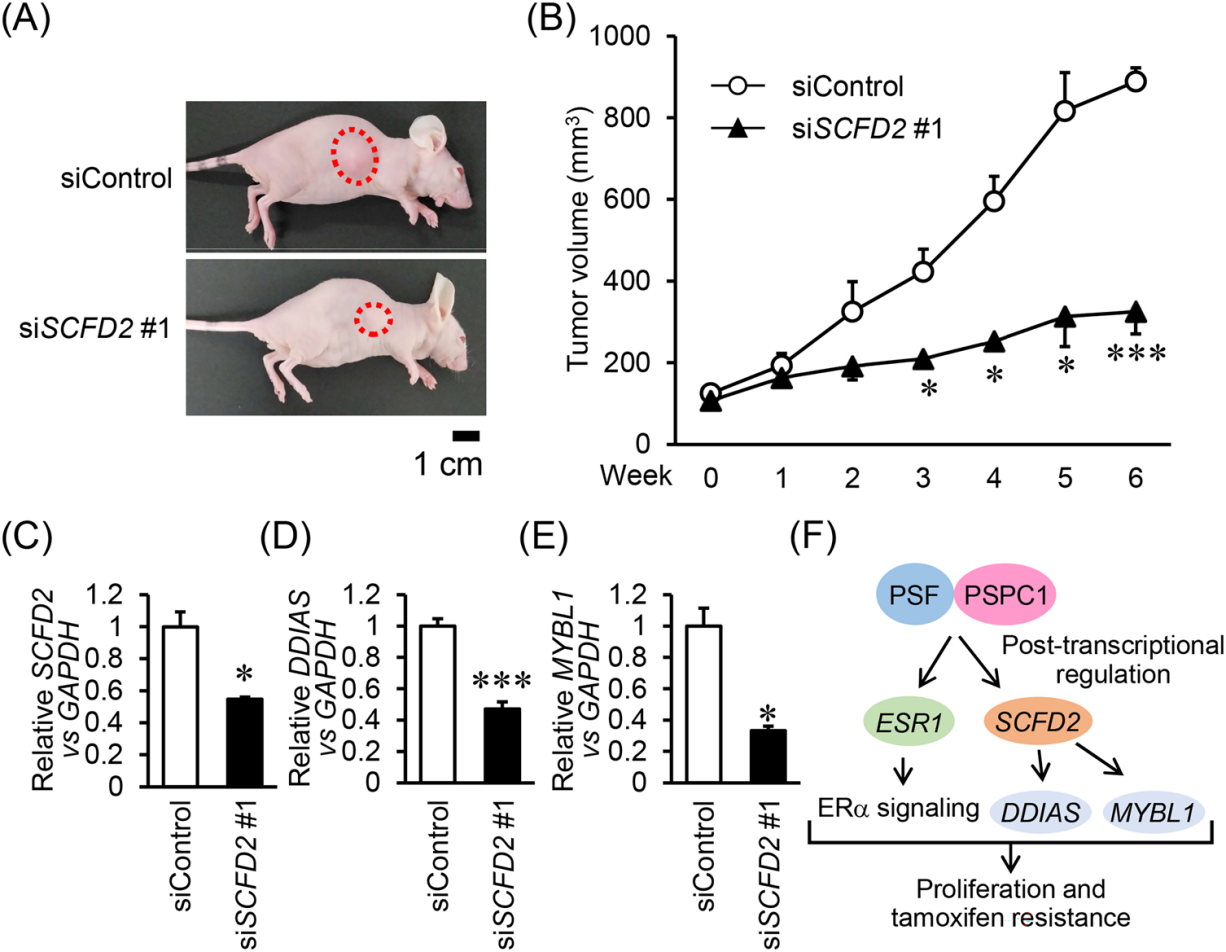
SCFD2-targeted siRNA suppresses in vivo tumor growth of tamoxifen-resistant breast cancer cells. (**A**) Representative images of xenografted mice at the day of sacrifice. (**B**) Effect of si*SCFD2* #1 on growth of OHTR-derived xenograft tumors in athymic mice. Indicated siRNAs were intratumorally injected into xenografted mice twice a week. Tumor volumes are presented as mean ± SEM (*n* = 4). (**C**–**E**) Expression of *SCFD2* (**C**), *DDIAS* (**D**), and *MYBL1* (**E**) mRNAs in OHTR-derived tumors. Relative expression levels of these mRNAs were normalized to *GAPDH* mRNA level in each sample and presented as mean fold ± SEM compared with siControl (*n* = 4). *P < 0.05; ***P < 0.001, Student’s *t*-test. (**F**) Schematic image for a potential role of PSPC1/PSF/SCFD2 axis in ER-positive breast cancer.

Finally, we examined the role of SCFD2 in in vivo tumor growth of tamoxifen-resistant breast cancer cells. To this end, OHTR cells were xenografted to female athymic mice and an siRNA against SCFD2 was administrated into generated tumors. As a result, si*SCFD2* #1 suppressed OHTR tumor growth (Fig. 5A,B). *SCFD2* was efficiently downregulated by si*SCFD2* #1 (Fig. 5C). Moreover, *DDIAS* and *MYBL1* mRNA expression was downregulated by administration of si*SCFD2* #1 (Fig. 5D,E). These results support the findings of our in vitro experiments and indicated the significance of SCFD2 in vivo.

## Discussion

In the present study, we showed that PSPC1 post-transcriptionally regulates *ESR1* and *SCFD2* and potentially contributes to the proliferation of ER-positive breast cancer cells. PSPC1 interacted with PSF and transcripts of *ESR1* and *SCFD2* genes in MCF-7 and OHTR cells. Moreover, PSPC1 silencing caused a decrease in amount of *ESR1* and *SCFD2* mRNAs but not in those of intron region, suggesting that PSPC1 positively affects splicing process of intron-containing mRNA precursors. We have previously reported that siRNA-mediated suppression of PSF increases or has no effect on intron levels in *ESR1* and *SCFD2* genes^12,17^. In addition, the double silencing of PSPC1 and PSF potently suppressed the growth of MCF-7 and OHTR cells than single silencing of PSPC1. Thus, PSPC1 and PSF will cooperatively increase the expression levels of their target genes through post-transcriptional regulation.

We previously reported that *SCFD2* is a PSF target and promotes the proliferation and tamoxifen resistance of ER-positive breast cancer cells^12^. In the present study, we revealed that strong SCFD2 IR was a potential prognostic marker for ER-positive breast cancer patients, and the combinations of strong intensities of PSPC1, PSF, and SCFD2 IRs were more potently associated with poor prognosis in these patients.

Notably, we further showed *DDIAS* and *MYBL1* as downstream target genes of SCFD2 in ER-positive breast cancers based on transcriptomic analysis. DDIAS is an anti-apoptotic factor that suppresses cell death caused by various DNA-damaging stresses such as cisplatin treatment and UV irradiation^18–20^. Recently, it was demonstrated that DDIAS inhibits the death-inducing signaling complex (DISC) formation and promotes destabilization of caspase-8, thus suppressing TRAIL-mediated apoptosis^21^. DDIAS is also involved in homologous recombination DNA repair and IL-6/STAT3 signaling activation, which may contribute to cancer progression^22,23^. Immunohistochemical analyses showed that DDIAS expression was associated with shorter survival of breast cancer and non-small-cell lung cancer patients^24,25^. DDIAS regulates cell cycle progression in a context-dependent manner, as it promotes G1 to S phase transition in HCC cells^20^ whereas hardly affects cell cycle progression in osteosarcoma U2OS-SCR cells^22^. Interestingly, there is a report that PSPC1 knockdown increased apoptosis in HeLa cells treated with DNA-damaging agent methyl methanesulfonate^26^. Considering that DDIAS expression is induced by DNA damage response in cancer^19^, PSPC1 and SCFD2 may contribute to the suppression of apoptosis partly dependent on DDIAS.

MYBL1 is a MYB family transcription factor and its gene amplification and rearrangements were observed in some cancers^27–31^. *MYBL1* high expression was shown to be associated with shorter survival of HCC and those with ER-positive breast cancer^32,33^. In HCC, MYBL1 is shown to promote cancer cell proliferation by activating *TWIST1* transcription^32^. It was also suggested that MYBL1 rescues murine B-cell lymphoma cells form the growth arrest and apoptosis induced by anti-immunoglobulin M (IgM) antibody through maintaining *c-Myc* expression^34^. Further study on the function of DDIAS and MYBL1 will be useful to elucidate the role and mechanism of SCFD2 in ER-positive breast cancer progression.

Finally, we demonstrated that siRNA-mediated *SCFD2* silencing suppressed in vivo tumor growth of tamoxifen-resistant breast cancer cells, suggesting that SCFD2 can be a promising therapeutic target of tamoxifen-resistant breast cancer.

In conclusion, the present results suggest that PSPC1 would facilitates hormone-dependent breast cancer proliferation by exhibiting RNA processing of *ESR1* and *SCFD2*, the latter further contributes to anti-apoptotic or proliferative responses by modulating the expression of *DDIAS* and *MYBL1* (Fig. 5F).

## Methods

### Cell culture and siRNA transfection

Human ER-positive breast cancer MCF-7 cells and its 4-hydroxytamoxifen (OHT)-resistant OHTR cells were previously described^12,15^. Cell authentication was confirmed by STR profiling. Cells were transfected with siRNAs using Lipofectamine RNAiMAX reagent (Thermo Fisher Scientific, Waltham, MA, USA) according to the manufacturer's instructions. siRNAs used in this study are listed in Supplementary Table S2.

### Quantitative real-time polymerase chain reaction (qRT-PCR)

RNA was extracted from MCF-7 or OHTR cells using ISOGEN reagent (Nippon gene, Tokyo, Japan) according to the manufacturer’s protocol. For tumors generated from OHTR cells xenografted to nude mice, RNA extraction was performed using Sepasol-RNA I Super G (Nacalai Tesque, Kyoto, Japan). RNA was reverse-transcribed with SuperScript III reverse transcriptase (Thermo Fisher Scientific) or PrimeScript RT reagent Kit (Perfect Real Time) (Takara Bio, Shiga, Japan). qRT-PCR was performed on StepOnePlus Real-Time PCR System (Thermo Fisher Scientific) using KAPA SYBR FAST qPCR Kit (KAPA Biosystems, Wilmington, MA, USA). mRNA expression level was evaluated by qRT-PCR and normalized to *GAPDH* mRNA level in each cell sample^12^. Primer pairs used in this study are listed in Supplementary Table S3.

### Western blotting

Whole cell lysates were prepared from MCF-7 or OHTR cells transfected with indicated siRNAs for 72 h. Western blotting using anti-PSPC1 (sc-374181, Santa Cruz Biotechnology, Dallas, TX, USA), β-Actin (A2228, Sigma-Aldrich, St. Louis, MO, USA), c-Myc (sc-789, Santa Cruz Biotechnology), and FLAG (F3165, Sigma-Aldrich) antibodies and subsequent detection were performed as previously described^12^.

### DNA assay

MCF-7 or OHTR cells were collected 1, 3, and 5 days after siRNA transfection. For quantification of cell proliferation, extracted DNAs were stained with 5 μg/mL of Hoechst 33258 Pentahydrate (bis-benzimide) (Thermo Fisher Scientific), and DNA contents were measured by 2030 ARVO X Multilabel Plate Reader (Perkin Elmer, Waltham, MA, USA)^12^.

### Cell cycle analysis

Flow cytometric analysis of cell cycle was performed as previously described^12^. Briefly, cells were harvested and fixed with 70% ethanol at − 30 °C overnight. Fixed cells were treated with RNase A and stained with 10 μg/mL propidium iodide (PI). These cells were analyzed using BD LSRFortessa (BD Biosciences, San Jose, CA). The percentage of cells in G0/G1-, S-, and G2/M-phases was evaluated using FlowJo software (BD Biosciences).

### Clinical samples and data

Clinical samples were obtained from 114 Japanese female ER-positive breast cancer patients who underwent surgical treatment between 2006 and 2013 at Toranomon Hospital (Tokyo, Japan). Patients’ ages ranged from 33 to 76 years, and none of the clinical samples received preoperative chemotherapy or targeted therapies. Treatment was carried out according to the National Comprehensive Cancer Network’s guidelines^35^. Staging was determined based on the TNM Classification of Malignant Tumors^36^. Follow-up duration ranged from 22 to 118 weeks, with an 86-week average.

### Immunohistological analysis

Immunohistochemical analyses were performed as previously described^37^. Briefly, 4-μm tissue sections were deparaffined with xylene, hydrated stepwise with ethanol, and then washed with TBS. Subsequently, the sections were heated in 10 mM sodium citrate buffer (pH 6.0) at 121 °C for 5 min for PSPC1 staining or 20 min for SCFD2 staining. Anti-PSPC1 (sc-374181, Santa Cruz Biotechnology) or SCFD2 antibody (HPA036526, Sigma-Aldrich) was applied to the sections at 1:100 dilution and incubated at 4 °C overnight. After washing with TBS, the sections were incubated with EnVision + HRP-labeled polymerase (Dako, Carpinteria, CA, USA) for 1 h at room temperature. DAB substrate kit (Vector Laboratories, Burlingame, CA, USA) was used for detection. For encapsulation, Permount (Thermo Fisher Scientific) was used. PSPC1 and SCFD2 immunoreactivities (IRs) were evaluated in the nucleus and the cytoplasm of carcinoma cells, respectively. Samples with > 1% and > 10% positive carcinoma cells were considered as tumors with strong IR of PSPC1 and SCFD2, respectively.

### Plasmid construction and transfection

To generate an expression vector encoding Myc-tagged PSPC1 (Myc-PSPC1), human PSPC1 cDNA lacking the first methionine was inserted into *Eco*RI-*Xho*I sites of mammalian expression plasmid pcDNA3.1(+) (Thermo Fisher Scientific), which was modified to generate the N-terminally Myc-tagged protein. An expression vector encoding FLAG-tagged human PSF (FLAG-PSF) was previously generated using pcDNA3 (Thermo Fisher Scientific)^38^. The transfection of these expression vectors and control vectors encoding only Myc- or FLAG-tag was performed 24 h after cell plating using FuGENE HD (Promega, Madison, WI, USA), according to the manufacturer’s protocols.

### Immunoprecipitation (IP) assay

MCF-7 and OHTR cells transfected with indicated plasmids were washed twice with PBS, and then harvested and lysed in IP buffer with the following formula: 50 mM Tris–HCl pH 7.5, 150 mM NaCl, 1% Nonidet P-40, and Protease Inhibitor Cocktail (EDTA free) (Nacalai Tesque). Cell lysates were centrifuged at 14,000 rpm for 15 min at 4 °C and protein concentrations of the collected supernatants were determined using Pierce BCA Protein Assay Kit (Thermo Fisher Scientific). For immunoprecipitation, 1 mg of lysates were mixed with Pierce Anti-c-Myc Agarose (Thermo Fisher Scientific) or ANTI-FLAG M2 Affinity Gel (Sigma-Aldrich) and rotated for 3 h at 4 °C. After antibody-conjugated beads were washed three times with IP buffer, precipitated proteins were eluted by 2** × **SDS sample buffer and analyzed by Western blotting.

### RNA-immunoprecipitation (RIP) assay

Cells were cross-linked with formaldehyde at a final concentration of 0.3% for 5 min and quenched by glycine at a final concentration of 36 mM for 5 min at room temperature. After washing with PBS, cells were scraped and resuspended in hypotonic buffer (10 mM HEPES pH7.9, 10 mM KCl, 0.1 mM EDTA, 0.1 mM EGTA, 0.6% NP-40, 1 mM DTT, 1 mM PMSF, and 3 μg/mL of aprotinin). Nuclear pellets were collected by the centrifugation at 4 °C, 15,000 rpm for 15 min, and then resuspended in RIP buffer (150 mM KCl, 25 mM Tris–HCl pH 7.4, 5 mM EDTA, and 0.5% NP-40) and lysed by 10 strokes with a 26G needle. Nuclear extracts were centrifuged at 15,000 rpm for 15 min at 4 °C, and the supernatants were mixed with anti-PSPC1 antibody or control mouse IgG and rotated overnight at 4 °C. PSPC1-RNA complexes were precipitated using Protein G Sepharose 4 Fast Flow (Cytiva, Marlborough, MA, USA), and bead-conjugated RNAs were eluted using Sepasol-RNA I Super G (Nacalai Tesque).

### Microarray and database analysis

To examine the gene expression of siRNA-treated MCF-7 cells, microarray analysis was performed using GeneChip Human Gene 1.0 ST Array (Thermo Fisher Scientific) according to the manufacturer’s protocol. Gene ontology (GO) analysis was performed using AmiGO2 database (http://amigo.geneontology.org/amigo). The Oncomine™ Platform (https://www.oncomine.org) was used to analyze gene expression in normal breast tissues, invasive ductal breast carcinoma (IDC), and invasive lobular breast carcinoma (ILC). For analyses of association between gene expression and relapse-free survival of ER-positive breast cancer patients, Kaplan–Meier Plotter software (https://www.kmplot.com) was used.

### Mice

BALB/cAJcI-nu/nu mice were purchased from CREA Japan (Tokyo, Japan). Animal care and experimental procedures were performed at animal facility of Tokyo Metropolitan Institute of Gerontology, and mice were maintained under a 12-h light–dark cycle (light on at 8:00 a.m.). All animal experiments were approved by the ethics committee of animal experiments at the Tokyo Metropolitan Institute of Gerontology (approval no. 18019) and performed in accordance with the animal experimental guidelines of the Tokyo Metropolitan Institute of Gerontology. This study complied with ARRIVE guidelines (https://arriveguidelines.org/).

### In vivo tumor formation and siRNA administration

OHTR cells (1** × **10^6^ cells) were mixed with the equal volume of Matrigel matrix (Corning, Corning, NY, USA) and injected subcutaneously into the side flank of 10-weeks-old BALB/cAJcI-nu/nu mice (CREA Japan, Tokyo, Japan). When the tumor volume reached 100 mm^3^, mice were divided randomly into two groups. siControl or si*SCFD2* #1 (5 μg each) was prepared with GeneSilencer Reagent (Genlantis, San Diego, CA, USA) and injected into the generated tumors twice a week. The tumor volumes were measured once a week as previously described^12^.

### Statistical analysis

Statistical analysis was performed using JMP version 9.0.0 (SAS Institute, Cary, NC, USA). Kaplan–Meier curves were generated using JMP, and *P* values were evaluated by log-rank test. For multiple comparisons, significance is assessed at *P* < 0.05 following Bonferroni correction. The correlation between PSPC1 or SCFD2 intensity scores and clinicopathological factors was evaluated by the Student’s *t*-test or Pearson’s chi-squared (χ^2^) test. Univariate and multivariate analyses were performed using a Cox proportional hazard model. For statistical analyses in studies using cultured breast cancer cells, *P*-values were evaluated by Student’s *t*-test or two-way ANOVA.

### Ethics statement

This study abided by the Declaration of Helsinki principles and was approved by the ethical committee of Toranomon Hospital (approval no. 845 and 1327) and Saitama Medical University International Medical Center institutional review board (approval no. 17-024).

## Supplementary Information

Supplementary Information.

## Data Availability

Microarray data are deposited in the Gene Expression Omnibus (GEO) database with the accession number GSE163553 and are available from the web link as below: [https://www.ncbi.nlm.nih.gov/geo/query/acc.cgi?acc=gse163553].

## Abbreviations

ANOVA: Analysis of variance
AR: Androgen receptor
BMI: Body mass index
DAB: 3,3′Diaminobenzidine
DBHS: *Drosophila* behavior human splicing
DDIAS: DNA damage induced apoptosis suppressor
DISC: Death-inducing signaling complex
DMEM: Dulbecco’s modified Eagle’s medium
EMT: Epithelial–mesenchymal transition
ER: Estrogen receptor
ESR1: Estrogen receptor 1
GAPDH: Glyceraldehyde-3-phosphate dehydrogenase
GEO: Gene expression omnibus
GO: Gene ontology
HCC: Hepatocellular carcinoma
HER2: Human epidermal growth factor receptor 2
IDC: Invasive ductal breast carcinoma
IL-6: Interleukin-6
ILC: Invasive lobular breast carcinoma
IP: Immunoprecipitation
IR: Immunoreactivity
LVI: Lymphovascular infiltration
MYBL1: MYB proto-oncogene like 1
PBS: Phosphate-buffered saline
PR: Progesterone receptor
PSF: Polypyrimidine tract-binding protein-associated splicing factor
PSPC1: Paraspeckle component 1
qRT-PCR: Quantitative real-time polymerase chain reaction
RBP: RNA-binding protein
RIP: RNA-immunoprecipitation
SCFD2: Sec1 family domain-containing 2
SDS: Sodium dodecyl sulfate
STAT3: Signal transducer and activator of transcription 3
STR: Short tandem repeat
TBS: Tris-buffered saline
TCGA: The Cancer Genome Atlas
TRAIL: Tumor necrosis factor-related apoptosis-inducing ligand
4-OHT: 4-Hydroxytamoxifen
