# Supplementary material for: PSPC1 is a potential prognostic marker for hormone-dependent breast cancer patients and modulates RNA processing of ESR1 and SCFD2
Source: Sci Rep. 2022 Jun 9;12:9495. doi: 10.1038/s41598-022-13601-7 (PMC9184599; doi:10.1038/s41598-022-13601-7)
Supplement: Supplementary file 1 — Supplementary Information. [file 41598_2022_13601_MOESM1_ESM.pdf]

Supplementary Information

PSPC1 is a potential prognostic marker for hormone-dependent breast cancer patients and modulates RNA processing of *ESR1* and *SCFD2*

Toshihiko Takeiwa, Kazuhiro Ikeda, Takashi Suzuki, Wataru Sato, Kaori Iino, Yuichi Mitobe, Hidetaka Kawabata, Kuniko Horie & Satoshi Inoue

**Supplementary Table S1.**	**GO analysis of TOP 150 genes whose expression was downregulated by SCFD2 knockdown.**
**Supplementary Table S2.**	**siRNAs used in this study.**
**Supplementary Table S3.**	**Primers used in qRT-PCR experiments.**
**Supplementary Figure S1.**	**Knockdown of *PSPC1* and *PSF* markedly impaired the proliferation of ER-positive breast cancer cells compared with knockdown of *PSPC1* alone.**
**Supplementary Figure S2.**	**Heatmaps of genes downregulated by SCFD2 knockdown in MCF-7 cells, related to Supplementary Table S1.**
**Supplementary Figure S3.**	**Unprocessed original images of Western blots.**
**Supplementary Figure S4.**	**Original flow cytometry data, related to Fig. 1G,H.**

Supplementary Table S1.
GO analysis of TOP 150 genes whose expression was downregulated by SCFD2 knockdown.

Term	Count	*P* value	Genes
Cell cycle process	24	6.26E-3	*PRIM1, DSCC1, XRCC2, ORC3, CLSPN, CCNE2, CENPK, MIS12, NUP43, MYH9, CDC25A, NABP1, CENPI, E2F7, POGZ, ATAD5, NCAPH, ANLN, NEK7, CRLF3, SKIL,* ***MYBL1****, MASTL,* ***DDIAS***
Cell cycle	27	1.78E-2	*PRIM1, PDCD2L, DSCC1, XRCC2, ORC3, CLSPN, CCNE2, CENPK, MIS12, NUP43, MYH9, CDC25A, NABP1, CENPI, E2F7, POGZ, ATAD5, NCAPH, ANLN, BRCC3, NEK7, CRLF3, LMLN, SKIL,* ***MYBL1****, MASTL,* ***DDIAS***
Mitotic cell cycle	19	2.80E-2	*PRIM1, DSCC1, XRCC2, ORC3, CCNE2, CLSPN, CENPK, MIS12, NUP43, CDC25A, NABP1, CENPI, E2F7, POGZ, NCAPH, ANLN, CRLF3,* ***MYBL1****, MASTL*

Supplementary Table S2.
siRNAs used in this study.

siRNA		Sequence (5' to 3')
si*PSPC1* #1	Sense strand	GAGGCUCUUCGAACGCUAUGG
	Antisense strand	AUAGCGUUCGAAGAGCCUCUU
si*PSPC1* #2	Sense strand	GAGCUGCUAGAGCAAGCAUUU
	Antisense strand	AUGCUUGCUCUAGCAGCUCAU
si*SCFD2* #1	Sense strand	GAGCAUCCGCUAAGAAGAUAA
	Antisense strand	AUCUUCUUAGCGGAUGCUCAG
si*SCFD2* #2	Purchased from Thermo Fischer Scientific	(Catalog number: s45714)
si*PSF #1*	Sense strand	GGCACGUUUGAGUACGAAUAU
	Antisense strand	AUUCGUACUCAAACGUGCCAU
si*PSF* #2	Purchased from Thermo Fischer Scientific	(Catalog number: HSS109643)
siControl	Sense strand	GUACCGCACGUCAUUCGUAUC
	Antisense strand	UACGAAUGACGUGCGGUACGU

Supplementary Table S3.

Primers used in qRT-PCR experiments.

Gene name	Forward (5' to 3')	Reverse (5' to 3')
GAPDH	ggtggtctcctctgacttcaaca	gtggtcgttgagggcaatg
PSPC1	gggcgagaagacgtacacg	aagcgaatccgtagaggtctg
ESR1 mRNA	agacggaccaaagccacttg	ccccgtgatgtaatacttttg
ESR1 intron	cctttgccttcagctaccag	gccataatcctctgggaaca
SCFD2 mRNA	aaacacccacagactgccaa	aacggacattgctgactccc
SCFD2 intron	agccatgtttccagcatcca	acagagtcccttggtactgga
DDIAS	aggttcagatgccagtaacttct	agtgattgttaggtgcctgaga
MYBL1	aggcaagcagtgtagagaaaga	cgatttcccaaccgcttatgt

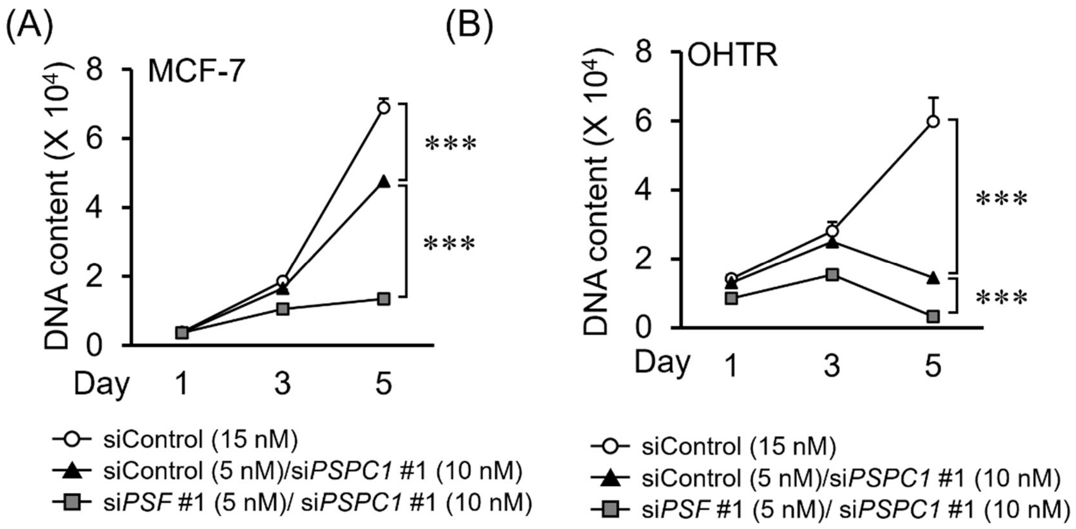

Supplementary Figure S1. Knockdown of *PSPC1* and *PSF* markedly impaired the proliferation of ER-positive breast cancer cells compared with knockdown of *PSPC1* alone. Proliferation of MCF-7 (A) and OHTR (B) cells treated with the indicated siRNAs at the indicated concentration was analyzed by DNA assay. Data are presented as mean value ± SEM (n = 5). ***, P < 0.001; Student's t-test.

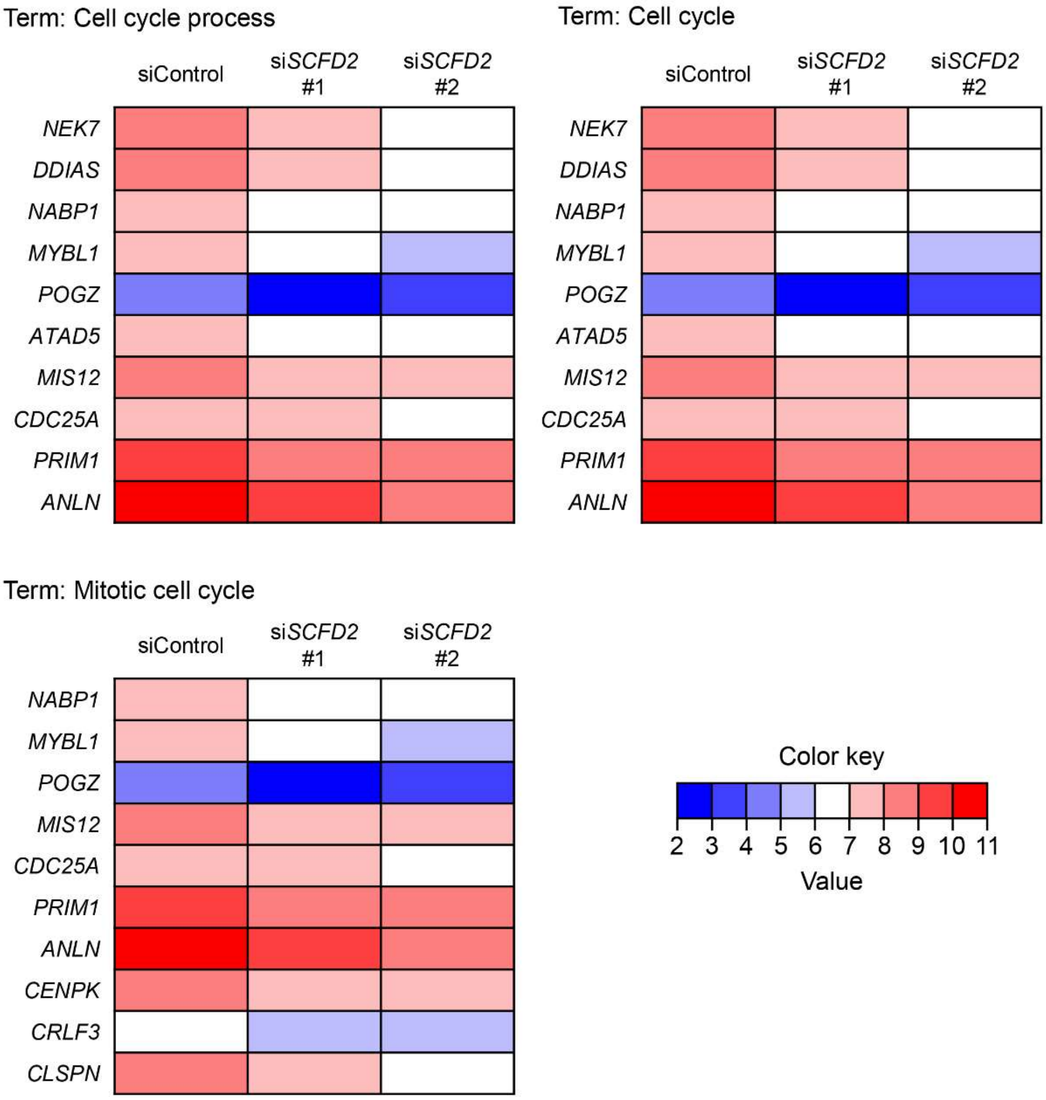

Supplementary Figure S2. Heatmaps of genes downregulated by SCFD2 knockdown in MCF-7 cells, related to Supplementary Table S1. The top 10 downregulated genes in each gene ontology (GO) term shown in Supplementary Table S1 are presented in heatmaps. Gene expression color key is shown in log_2 scale.

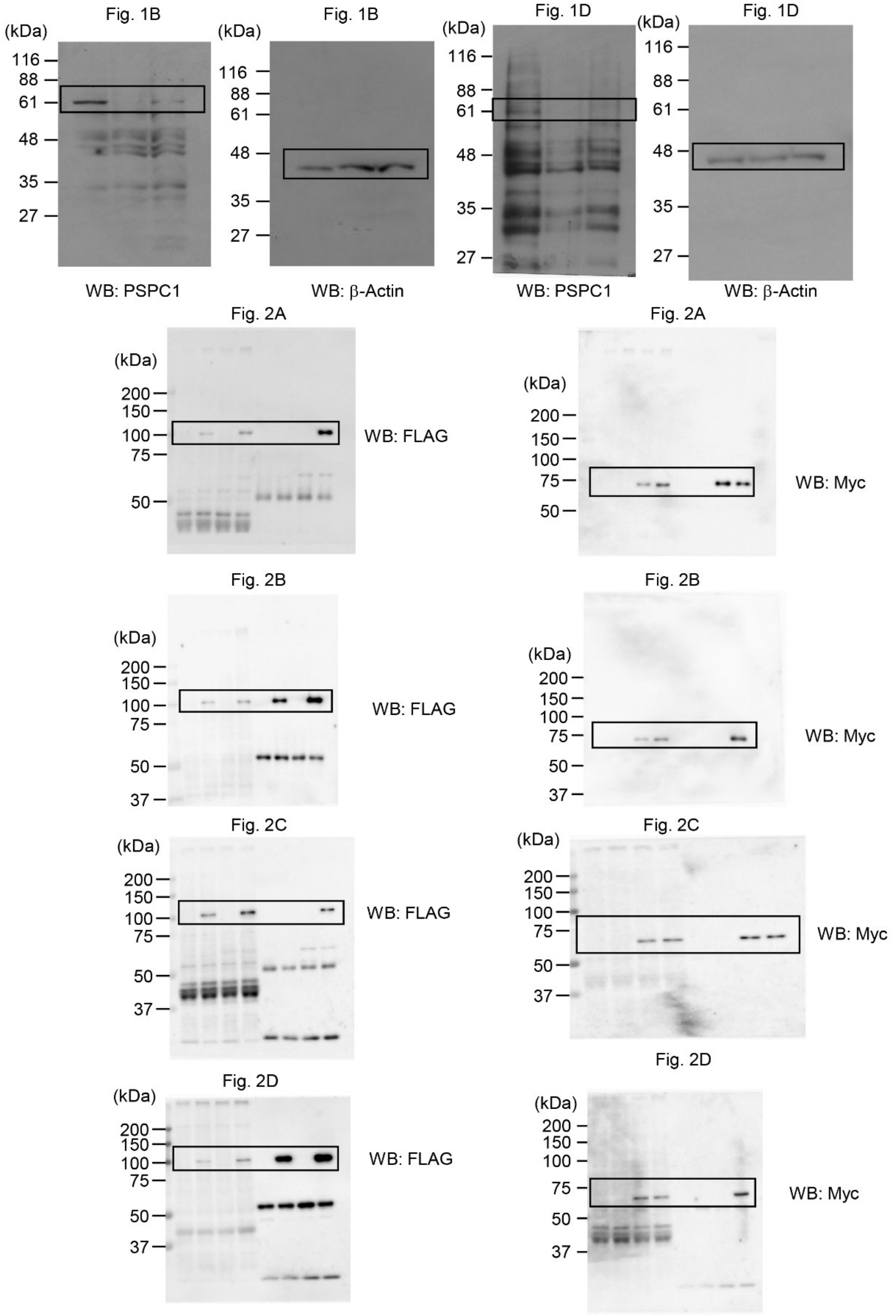

Supplementary Figure S3. Unprocessed original images of Western blots.

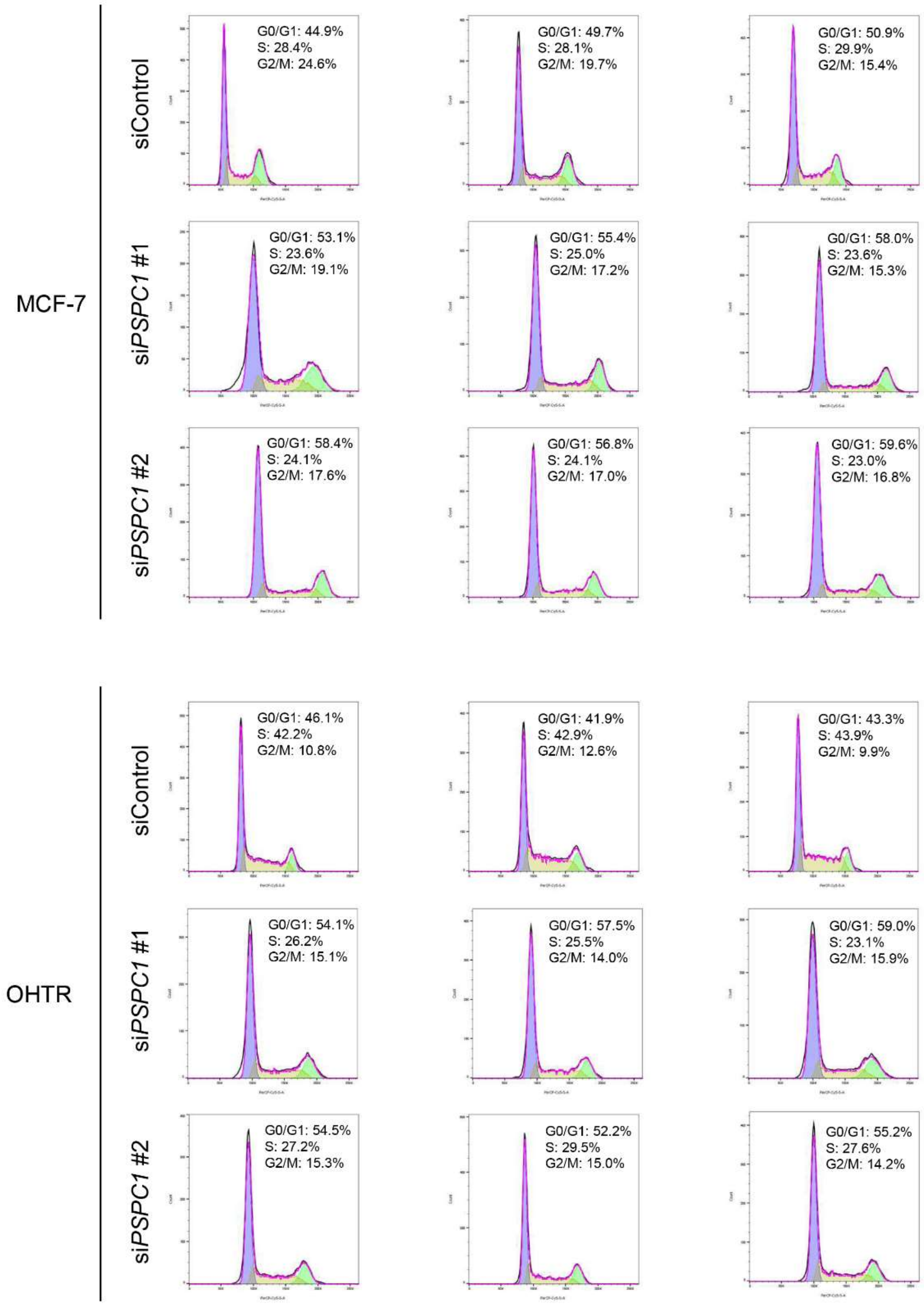

Supplementary Figure S4. Original flow cytometry data, related to Fig. 1G,H. Propidium iodide (PI) histograms obtained from flow cytometric analysis of MCF-7 and OHTR cells transfected with indicated siRNAs. The proportion of cells in the G0/G1 phase (blue area), S phase (yellow area), and G2/M phase (green area) of the cell cycle was quantified by FlowJo software.
